# ISG15 Promotes Progression and Gemcitabine Resistance of Pancreatic Cancer Cells Through ATG7

**DOI:** 10.7150/ijbs.85424

**Published:** 2024-01-21

**Authors:** Yiling Meng, Lei Bian, Meichao Zhang, Pingting Zhou, Suning Zhang, Yingxia Ying, Sunhu Yang, Yuanhua Liu, Yuan Yao, Dong Li

**Affiliations:** 1 Department of Radiation Oncology, Shanghai Ninth People's Hospital, Shanghai Jiaotong University School of Medicine, Shanghai, China.; 2 State Key Laboratory of Oncology in South China, Guangdong Provincial Clinical Research Center for Cancer, Sun Yat-sen University Cancer Center, Guangzhou, China.; 3 Department of Emergency, Shanghai Ninth People's Hospital, Shanghai Jiaotong University School of Medicine, Shanghai, China.; 4 Department of General Surgery, Shanghai university of TCM Shanghai TCM-integrated hospital, Shanghai, China.; 5 Department of Chemotherapy, Nanjing Medical University Affiliated Cancer Hospital, Cancer Institute of Jiangsu Province, Nanjing, Jiangsu, China.

**Keywords:** pancreatic cancer, ISG15, autophagy, ATG7, Gemcitabine

## Abstract

Chemoresistance is an obstacle of improving pancreatic cancer (PC) prognosis. However, the biological function of ISG15 in PC and whether it correlates with the resistance to chemotherapy are still unknown. Here, we aimed to reveal the clinical significance of ISG15 in PC and its regulatory mechanism in cancer progression and resistance to therapy. The level of ISG15, a protein involved in post-translational modifications, is elevated in PC tissues. Clinically, higher ISG15 expression correlates with higher PC grades, stronger resistance to treatment and poorer prognosis. Moreover, ISG15 promotes the proliferation, migration, invasion, colony formation of PC cells and resistance to Gemcitabine, a classic chemotherapeutics for PC, both *in vitro* and *in vivo*. ISG15 promotes progression and resistance to therapy in PC cells by binding to ATG7, reducing its degradation, and thereby leading to enhanced autophagy in PC cells. ISG15 may be used as both a potential diagnosis marker and sensitizer for chemotherapeutics such as Gemcitabine during PC intervention.

## Introduction

Pancreatic cancer (PC) is one of the most lethal cancer types and has a 5-year survival rate of 9% 1. The extremely high mortality can be ascribed largely to the lack of early detection, which renders patients no longer accessible for surgical resection, while other treatments have nominal influences on survival 2. Nonsurgical therapies for PC include chemotherapy, radiotherapy and palliative care. Gemcitabine is considered as the standard chemotherapy in the last decades and has demonstrated moderate effects on the survival of PC patients 3. However, intrinsic or required resistance critically restricts its effectiveness with various contributing mechanisms 4. Thus, enhancing the efficacy of adjuvant therapy is one of the most important challenges in PC studies.

Autophagy is an intracellular catabolic process in which proteins and organelles are recycled using lysosomal degradation. Autophagy is reportedly closely related to cancer, but the exact role remains elusive or controversial for different cancer types or tumor stage 5-8. In PC, increasing evidence shows that autophagy may play a promoting role in PC tumorigenesis, metastasis and the development of resistance towards adjuvant therapy 9-11. Administration of chemotherapeutic drugs, such as Gemcitabine, can activate autophagy in PC cells 12-14. Inhibition of autophagy may confer sensitivity towards these chemotherapeutics 13, 15-17. Nonetheless, the results from a phase 2 randomized clinical trial in patients with advanced PC demonstrated that the addition of hydroxychloroquine (HCQ), an inhibitor of autophagy, to Gemcitabine in combination with nab-paclitaxel, cannot improve overall survival 18. These findings suggest that new ways of intervening autophagy such as effective sensitizers may be needed for the standard chemotherapy of PC.

Interferon-induced gene 15 (ISG15) is the first identified ubiquitin-like protein 19. Similar to ubiquitin conjugation, ISG15 conjugates (ISGylation) with target proteins and regulates protein homeostasis utilizing a three-step enzymatic cascade which involves several enzymes 20. ISG15 and ISGylation reportedly play a pivotal role in maintaining protein homeostasis by modulating ubiquitin-proteasomes and autophagy, although the function and mechanism remain elusive 21. In addition to its extensively studied role in anti-viral responses 22, ISG15 and its ISGylation are involved in the pathogenesis of various types of cancer 20, 23. In PC, ISG15 is critical for cancer stem cell (CSC) maintenance 24-26. It is found that tumor-associated macrophages in the tumor microenvironment of PC promoted the growth of cancer stem cells of PC by secreting ISG15, thereby enhancing the proliferation, self-renewal, and invasion of PC 24. However, the role of ISG15 in chemotherapy of PC is poorly studied. *Ina et al* identified ISG15 as the gene related to Gemcitabine sensitivity in vitro 27. It is also reported that the increased expression of ISG15 is related to the resistance of pancreatic cancer cells to gemcitabine 27. However, the precise mechanism and function of ISG15 and ISGylation in PC development and treatment remain elusive. Therefore, the dissection of the mechanisms by which ISG15 and ISGylation confer drug resistance may be beneficial in improving the efficacy of PC adjuvant therapy.

In this study, we found that ISG15 is highly expressed in PC tissues and promotes cell proliferation, metastasis and resistance to Gemcitabine both in vitro and in vivo. We further showed that ISG15 exerts its impact on PC cells by modulating autophagy. ISG15 binds to ATG7, an essential molecule for autophagy, and stabilizes ATG7 by preventing its degradation and ultimately promoting cellular autophagy. Together, our data suggest a previously unrecognized mechanism in which ISG15 modulates PC treatment sensitivity and highlights a potential new target for therapeutic intervention.

## Methods and Materials

### Bioinformatics analysis of ISG15 expression and survival

Datasets (GSE16515, GSE62452, GSE57495) from GEO database were used for ISG15 expression and survival analysis. Kaplan-Meier survival analysis 28 was used to reveal the correlation between ISG15 expression and the survival of pancreatic cancer.

### Human tissue microarray

**Figure 1:**
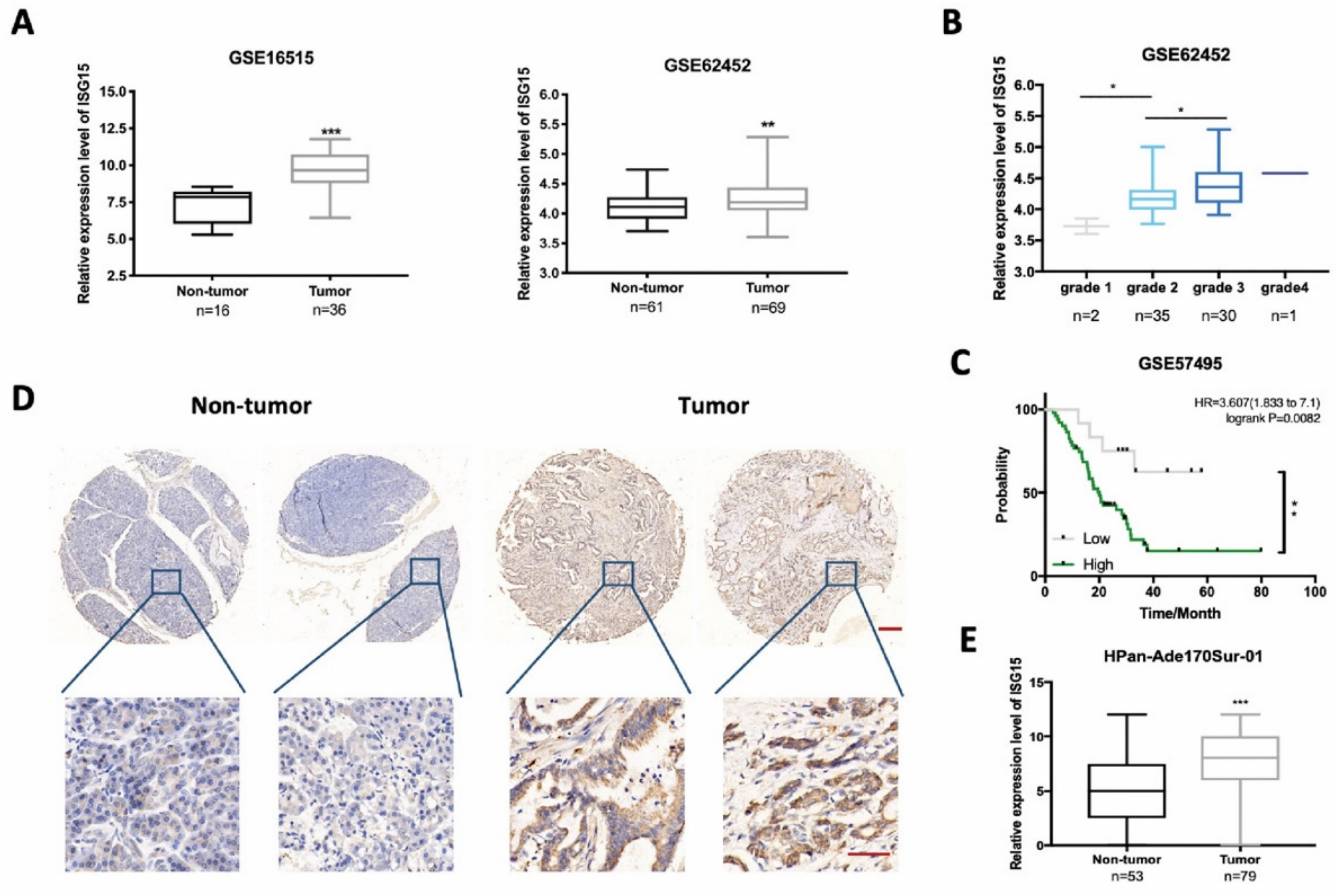
** ISG15 is highly expressed in pancreatic cancer (PC) tissues. A,** Bioinformatics analysis of ISG15 in PC tumor and non-tumor tissues using data from GSE16515 and GSE62452 with numbers enrolled (n) indicated on the bottom. **B,** Box-plot of the expression of ISG15 in PC tissues of different differentiation grades. **C,** Log-rank analysis of the correlation between ISG15 expression and survival of 63 PC patients using data from GSE57495. **D,** Representative images of ISG15 IHC staining in tumor and non-tumor tissues from PC microarray HPan-Ade170Sur-01 (scale bar; 50 μm) with zoomed-in pictures shown on the bottom (scale bar; 200 μm). **E,** Box-plot of the expression of ISG15 in tumor and adjacent non-tumor tissue sections from PC microarray. Method of H-score evaluation was described in Methods and Materials. (* *p*<0.05; ** *p*<0.01; *** *p*<0.001).

The tissue array that contains 69 primary pancreatic cancer tissues and 53 adjacent non-tumor tissues was purchased from Shanghai Xinchao Company. No enrolled patients had received preoperative treatment. The tissue sections were stained with antibody against ISG15 (1:50, A1182, ABclonal) and ATG7 (1:500, GB11399-1, Servicebio). In addition, there were two missing points in the ATG7 staining results, so they were deleted in subsequent analysis. The data shown in Figure 1 and Figure S1 was generated based on the average IHC scores (H-scores) given by two independent researchers. Briefly, H-scores are the score of density that multiplies the score of range. The score of density was given by following the criteria: 0 for negative expression; 1 for weak (light brown); 2 for moderate (yellow brown); and 3 for strong (brown), respectively. For the score of range: 1 for less than 10 % expression; 2, 11 to 30 %; 3, 31 to 75 % and 4, 76 to 100%, respectively.

### Cell lines and cell culture

Human PANC-1 and Mia Paca-2 were obtained from the cell bank of Chinese Academy of Science and cultured as recommended. Briefly, the two cell lines were cultured in Dulbecco's modified Eagle medium (DMEM) with 10% FBS, 100 U/ml penicillin, and 100 μg/ml streptomycin in a humidified incubator with 5% CO_2_ at 37 °C.

### Retroviral production and infection

Retroviral vectors containing non-target shRNAs or specific shRNAs targeting ISG15 were transfected into 293T cells using a transfection reagent (QIAGEN) following the manufacturers' instructions. To obtain stable cell lines, supernatants containing viruses were harvested 48-hour post transfection and used to infect target cells with polybrene (10 µg/ml), followed by selection with puromycin. Sequences for ISG15 knockdown are as follows:

sh1 5'-GCACCGTGTTCATGAATCT-3';

sh2 5'-CGGAAATAAAGGCTGTTGT-3',

with sh2 targeting the 3'-UTR.

### Cell viability and colony formation assay

Cells with or without indicated treatments were plated on 96-well plates. After incubation for the indicated time, cell proliferation was detected using CCK-8 kit (Dojindo Lab) following manufacturer's instruction.

In plate colony formation assay, cells were seeded onto 6-well plates, followed by various treatments, and were allowed 10-14 days of colony formation. Colonies formed were fixed by 4 % paraformaldehyde and stained with crystal violet. Colonies of >50 cells were counted as clonogenic survivors. Surviving fraction (SF) after certain treatments was calculated. SF(t)= [number of clonogenic survivor (t)] / [number of clonogenic survivors (c)], while t and c represent treatment and control, respectively.

### Wound healing, migration and invasion assays

Wound healing, migration and invasion assays were performed as described before 29. Briefly, for the wound-healing assay, the cells were cultured to the confluence of 90%, scratched by the tip of 200 μl pipettes, and followed with three days incubation without FBS and gap size was measured. For migration and invasion assays, the transwell filter chambers were pre-coated by with or without matrigel (BD Biosciences). 200 μl cell suspension with 5×10^4^ cells were seeded into the upper chamber, and incubated with 24-48h, the cells in the lower chamber were fixed and stained with 0.1% crystal violet.

### Tumor sphere formation assay

Tumor sphere formation assay were performed as described before 30. Briefly, the cells with a density of 10000 cells/ml were seeded in ultra-low attachment six-well plates and maintained in a medium with serum-free DMEM supplemented with B-27 (1:50; Invitrogen), 20 ng/ml bFGF (BD Biosciences), and 20 ng/ml EGF (BD Biosciences). The mammosphere number was counted and photographed.

### Western blotting

Cell pellets were lysed by lysis buffer and subjected for analysis. The samples were resolved by SDS-polyacrylamide gel electrophoresis and transferred onto a PVDF membrane (Merck Millipore). After blocked with 5% non-fat dry milk and immunoblotted with the appropriate primary and secondary antibody, the blot were measured by using the Fusion FX7 ECL western blot system (Vilber Lourmat). Antibodies used are as follows:

Anti-LC3 A/B (#12741), Anti-SQSTM1/p62 (#39749), Anti-ATG3 (#3415), Anti-ATG5 (#12994), Anti-ATG7 (#8558), Anti-ATG12 (#4180), Anti- β-actin (#3700) from Cell Signaling Technology, Anti-ISG15 (A1182) from ABclonal, Anti-FlAG (F3165) and Anti-HA (H6908) from Sigma Aldrich, and Anti-ISG15 (F-9) (sc-166755) used for ISGylation was from Santa Cruz Biotechnology.

### RT-PCR assay

According to the manufacturer's instructions, total cell RNA was isolated using TRIzol reagent (Takara), and transcribed to cDNA by SuperScript II reverse transcriptase (Invitrogen). The SYBR Green PCR Master Mix purchased from Applied Biosystems was used to perform Real-time PCR assay on an ABI PRISM Sequence Detector 7500 (PerkinElmer, Branchburg, NJ). The primer sequences for RT-PCR Assay are as follows:

### SiRNA transfection

According to the manufacturer's protocol, the transfection small interfering RNA (siRNA) obtained from GenePharma were carried out using Lipofectamine 3000 (Invitrogen). The sequences of siRNA oligonucleotides against ATG7 are as follows:

si-1 5'- CCAACACACUCGAGUCUUUTT -3';

si-2 5'- CCAACAUCCCUGGUUACAATT-3'.

### Flow cytometric analysis

Based on the manufacturer's protocol, cell apoptosis analysis was performed using Annexin V-FITC/PI kit (Yeasen) by FACS CantoTM flow cytometer. Cells were seeded onto 6-well plates, followed by a variety of treatments. Cell samples were collected and washed twice with ice PBS, then stained with Annexin V-FITC/PI reagents for 10-15 minutes in dark at room temperature.

### Autophagosome detection

LC3 is recognized as an autophagy marker. It goes through conversion from LC3A (LC3 I) to LC3B (LC3 II) and is located in autophagosomes. Thus, LC3 II symbolizes the formation of phagosomes. To visualize autophagosomes, GFP-mCherry-LC3 constructs were used, as previously described 31. GFP is quenched in lysosome, thus the green dots in unmerged pictures represent only autophagosomes while red dots represent both autophagosome and autolysosomes. In merged pictures, yellow and red dots represent autophagosomes and autolysosomes, respectively. Images were captured using a Nikon A1 confocal microscope.

### Ectopic expression and Co-Immunoprecipitation (Co-IP)

FLAG-tagged ISG15 (BC009507) and HA-tagged ATG7 (PPL00304-2f) were obtained from Public Protein/Plasmid Library. Plasmids were transfected into 293T cells using Lipofectamine 3000 (Invitrogen) and allowed 48 hours for expression. Cells were then harvested and lysed in IP lysis buffer (ThermoFisher Scientific) supplemented with proteinase inhibitor and phosphatase inhibitor cocktail (MCE). After lysis and centrifugation, cell lysates were subjected to immunoprecipitation with antibodies and magnetic beads (Merck Millipore) for overnight at 4 °C.

### Mouse experiments

All animal experimental procedures were approved by Animal Care and Use Committee of Shanghai Ninth People's Hospital affiliated to Shanghai JiaoTong University School of Medicine.

In total, twenty female nude Balb/c mice aged from 4 to 5 weeks old were purchased from Shanghai SLAC Laboratory Animal Co., Ltd. Mice were randomly divided into four groups (each group with 5 mice). On day 0, the group ISG15-NC+PBS and ISG15-NC+Gemcitabine received injection of 1×10^7^ ISG15-NC PANC-1 cells at the right flank of mice while the group ISG15-sh2+PBS and ISG15-sh2+Gemcitabine received injection of ISG15-sh2 PANC-1 cells. On day 7, 50 mg/kg of Gemcitabine (HY-B0003, MCE) dissolved in PBS was injected intraperitoneally for group ISG15-NC+Gemcitabine and ISG15-sh2+Gemcitabine while other group received injection of PBS of equal volume. Gemcitabine and PBS were given twice a week.

Tumor volumes were measured twice a week from day 7, and the average tumor volume was calculated using the equation: volume = (length × width^2^)/2.

Mice were sacrificed when any of the tumor volumes exceeded 1,000 mm^3^. Subcutaneous tumors were harvested and subjected to histological analysis using immunohistochemistry and TUNEL assays.

### Statistical analysis

All data were analyzed using GraphPad Prism software. Student's *t*-test or one-way ANOVA was performed to test differences between groups in both in vivo and in vitro experiments. Kaplan-Meier plots and Log-rank tests were used for survival analysis. Simple linear regression was used to analyze the relationship between ISG15 and ATG7 expression in PC microarray.* P* < 0.05 was considered statistically significant.

## Results

### High ISG15 expression is linked to poor differentiation and prognosis in PC patients

To compare the expression level of ISG15 between PC tissues and non-tumor tissues, we examined the Gene Expression Omnibus (GEO) database and excavated the data from two mRNA expression profiles (GSE16515 and GSE62452). We found that the expression level of ISG15 was significantly higher in PC tissues than in non-tumor tissues (Fig. 1A). A further analysis of dataset GSE62452 revealed that ISG15 expression was higher in poorly differentiated PC tissues (Grade 3-4) than well (Grade 1) or moderately differentiated (Grade 2) ones (Fig. 1B), but had no correlation with the tumor stage (Fig. S1A). The grade has been identified as a significant independent indicator of prognosis for PC patients after resection 32, 33. We then examined the correlation between ISG15 expression and prognosis. No significant difference of survival time was observed between PC and non-tumor tissues in dataset GSE62452 (p=0.0970) (Fig. S1B). However, the analysis of another dataset GSE57495 showed that high expression of ISG15 correlated with poor prognosis (Fig. 1C). Kaplan-Meier (K-M) survival analysis also supported these findings (Fig. S1C). Also, K-M survival analysis showed that patients with high expression level of ISG15 displayed poor recurrence-free survival (Fig. S1D). To further characterize ISG15 expression in PC, we examined ISG15 protein expression by using immunohistochemistry in 69 PC tissues and 53 adjacent normal tissues. The comparison of H-score of ISG15 expression between tumor and adjacent normal tissues showed that ISG15 expression was significantly higher in PC tissues (p<0.001) (Fig. 1D, E). ATG7 protein levels also is evaluated in tumor and non-tumor tissues from PC microarray, but the difference was not significant (Fig. S1E). Further analysis indicated a positive correlation between ISG15 and ATG7 in PC microarray (p<0.05) (Fig. S1F). These data suggest that ISG15 might be a regulator of malignant development in PC.

### ISG15 regulates malignant behaviors and responses to Gemcitabine in PC

**Figure 2:**
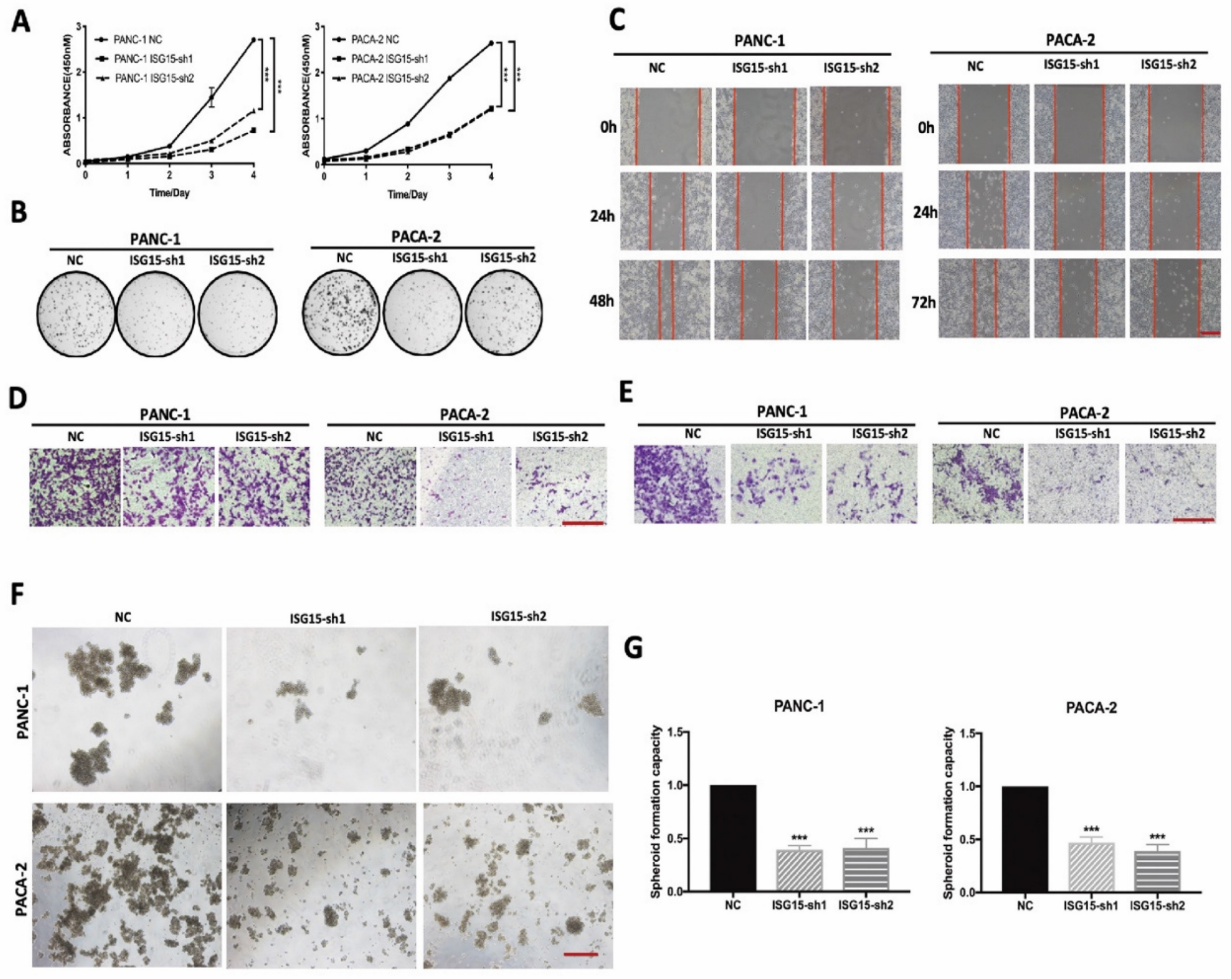
** ISG15 promotes malignant biological behaviors of PC cells. A,** Proliferation of PC cells with or without ISG15 depletion as determined by CCK-8 assay. **B,** Representative images of colony formation assays. **C,** Effects of ISG15 knockdown on cell migration capacity as valued by wound healing assay. Representative images are shown (scale bar; 100 μm). **D-E,** Representative images of chamber migration (D) and invasion assays (E) designated for evaluation of cell migration and invasion capacity, respectively (scale bar; 100 μm). **F-G,** Effects of ISG15 depletion on spheroid formation. Representative images are shown (F) with statistic results (G) (scale bar; 50 μm). (*** *p*<0.001).

To investigate the biological function of ISG15 in PC, we depleted ISG15 in PC cell lines by using shRNAs PANC-1 and MIAPaca2 (PACA-2) (Fig. S2A). We found that cell proliferation was significantly reduced in PC cells after ISG15 expression was downregulated (Fig. 2A). Similarly, experiments with colony formation assays showed that ISG15 downregulation resulted in significantly decreased number of colonies (Fig. 2B, Fig. S2B). Thus, ISG15 depletion inhibits the proliferation of PC cells in vitro.

We next assessed migration and invasion ability, another two essential capacities of PC cells. In experiments with the wound-healing assay to assess the migration activity of PC cells, we found that the cells with ISG15 depletion exhibited a more extensive wound closure area than cells without depletion (Fig. 2C, Fig. S2C). In addition, depletion of ISG15 significantly reduced the migration (Fig. 2D, Fig. S2D) and invasive (Fig. 2E, Fig. S2E) abilities of PC cells, as revealed by the Matrigel assay. At the same time, we investigated the role of ISG15 in maintaining the ability of spheroid formation and found that ISG15 depletion reduced the number of spheroids (Fig. 2F, G). Thus, ISG15 regulates the malignant behaviors of PC cells.

**Figure 3:**
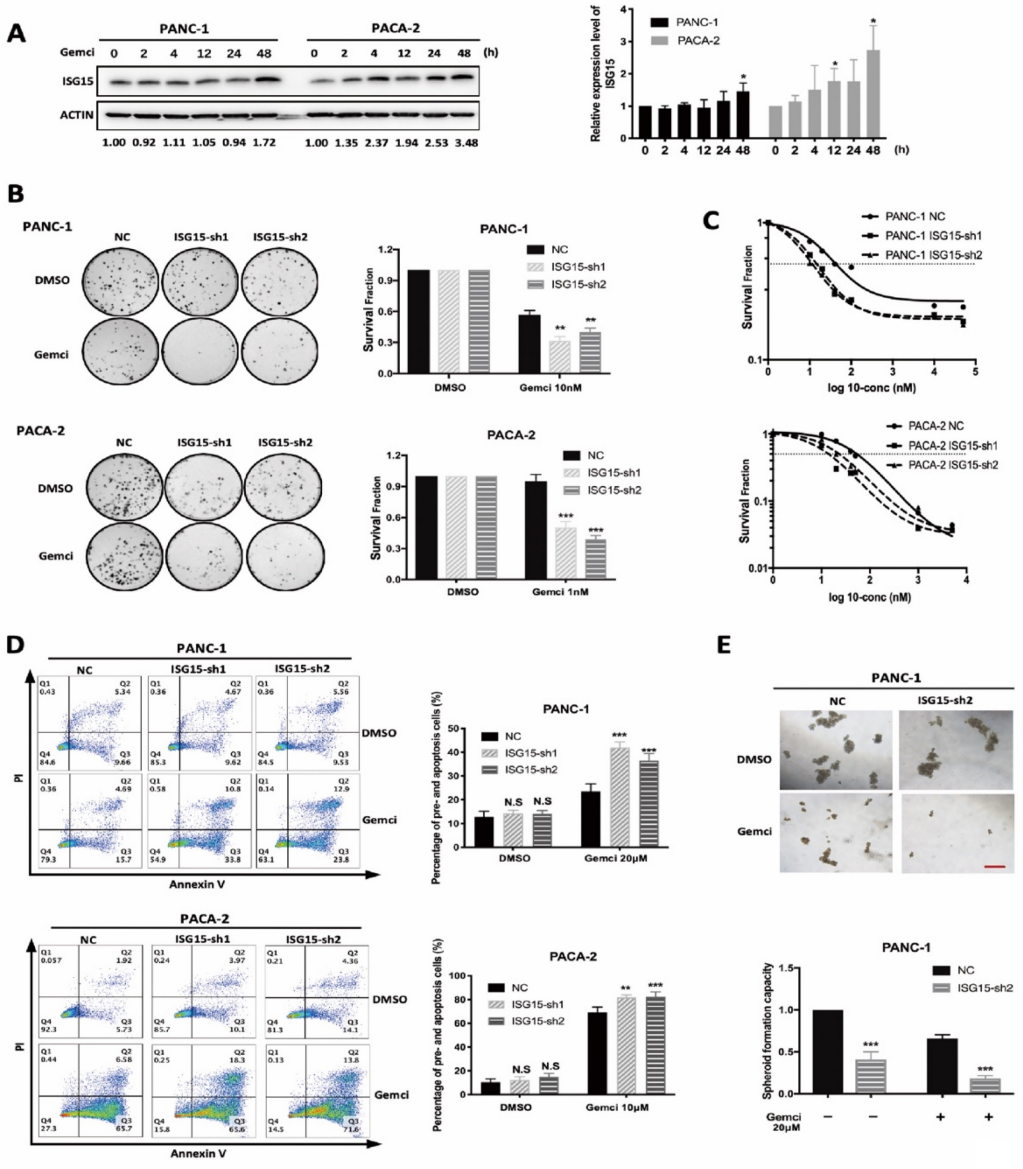
** ISG15 expression correlates with resistance to Gemcitabine. A,** ISG15 expression was elevated after Gemcitabine (Gemci) treatment. PC cells were treated with DMSO and Gemcitabine. After different times of treatment, cells were collected and lysed for Western blotting analysis. Grey value of ISG15 was detected and normalized by ACTIN. Relative expression level of ISG15 was calculated as normalized grey value of certain time point / normalized grey value of ISG15 at time 0. Representative results of Western blotting are shown on the left, and quantitative results of at least three independent results are on the right. **B-C,** Down-regulation of ISG15 increased PC cell sensitivity to Gemcitabine. **B,** In colony formation assays, 24h after cells were plated, Gemcitabine was added for additional 24-hour incubation and then removed for colony formation. Representative results of colony formation assays are shown on the left with quantitative results of at least three independent results shown on the right. For the concentration of Gemcitabine used in the assay of colony formation, we conducted preliminary experiments using PANC-1 and PACA-2 cells (data not shown), and chose 10nM Gemcitabine to treat PANC-1 cells, and 1nM Gemcitabine to treat PACA-2 cells. **C,** Survival fraction in different concentration of Gemcitabine was calculated using CCK-8 assay. 24h after cells were plated, different concentrations of Gemcitabine was added. 48h later, CCK-8 detection was performed. Survival fraction (SF) is calculated following the formula: SF= (absorbance at certain concentration) / (absorbance at control) while DMSO was used as control. Representative results of at least three independent experiments are shown. **D,** Knock-down of ISG15 increased PC cell apoptosis after Gemcitabine treatment. Representative results of flow cytometry analysis are shown on the left with quantitative results shown on the right. **E,** ISG15 down-regulation enhanced the inhibition of spheroid formation by Gemcitabine. Representative results of spheroid formation are shown on the top panel with quantitative result shown on the bottom (scale bar; 50 μm). (* *p*<0.05; ** *p*<0.01; *** *p*<0.001).

Next, we elucidated whether ISG15 regulates drug resistance in PC cells. First, we examined whether ISG15 expression could be altered with drug treatment. Indeed, after Gemcitabine treatment, the level of ISG15 was elevated in PC cell lines (Fig. 3A). We then explored the role of ISG15 in modulating Gemcitabine sensibility. In experiments with the colony formation assay, depletion of ISG15 led to significantly elevated sensitivity to Gemcitabine (Fig. 3B). Similar results were also obtained from the cell viability assay; IC50 was reduced in PC cells transfected with shRNA targeting ISG15 (Fig. 3C). In addition, analysis of apoptosis showed that the rate of cellular apoptosis after Gemcitabine administration was significantly enhanced after ISG15 knockdown in PC cells (Fig. 3D). Also, the spheroid formation capacity was significantly weakened in ISG15-depleted cells after Gemcitabine treatment. The administration of Gemcitabine combined with ISG15 knockdown sharply reduced the number of spheroids formed by PC cells (Fig. 3E). Taken together, these data suggest that ISG15 confers chemoresistance to PC cells.

### ISG15 promotes PC proliferation and resistance to gemcitabine in vivo

To further confirm the role of ISG15 in PC cell proliferation and sensitivity to Gemcitabine *in vivo*, we established PC models in Balb/c nude mice. The tumors were derived from PANC-1 cells with or without stable knockdown of ISG15 (ISG15-sh2 or ISG15-NC, respectively). One week after tumor cells injection, Gemcitabine or control (PBS solution) was provided to investigate the impact of ISG15 on chemotherapeutic sensitivity.

**Figure 4:**
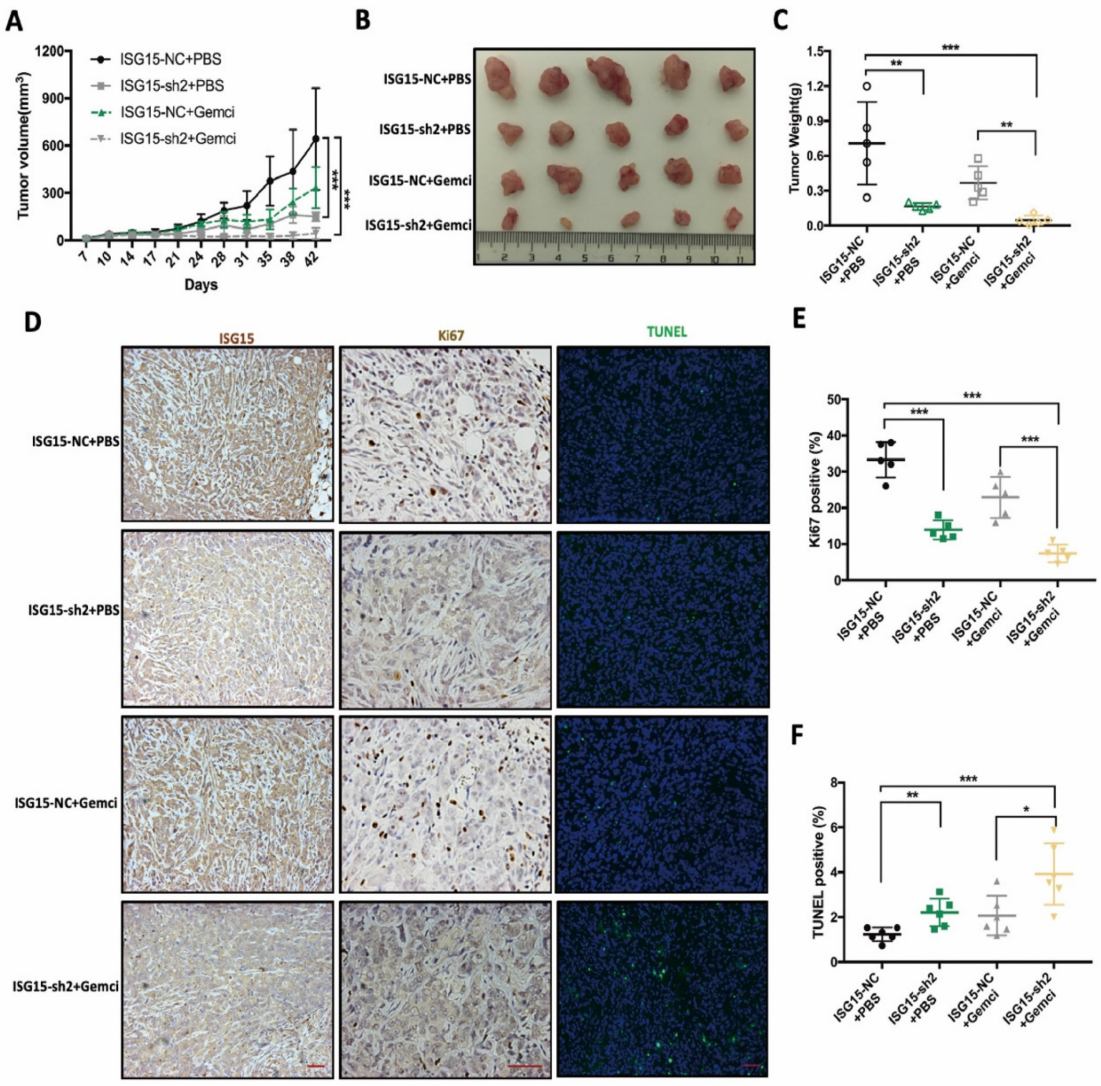
** ISG15 promotes tumor progression and Gemcitabine resistance *in vivo.* A,** Tumor size derived from ISG15-depleted or control PANC-1 cells with or without Gemcitabine treatment were measured in Balb/c nude mice. **B-C,** Tumor size (B) and weight (C) were measured after tumor excision at day 42. **D,** IHC staining of ISG15 and Ki67 and the results of TUNEL assays are shown (scale bar =50 μm). **E-F,** Statistic results of tumor weight (E) and the percentage of TUNEL-positive (apoptotic) cells (F) in PANC-1 derived tumors with different treatments. (* *p*<0.05; ** *p*<0.01; *** *p*<0.001).

We found that when compared with the control group (ISG15-NC+PBS), tumors with stable knockdown of ISG15 (ISG15-sh2+PBS) exhibited significantly slower growth (Fig. 4A). The average tumor size was significantly reduced in the ISG15-sh2+PBS group (Fig. 4B, C) compared with the control group.

While the administration of Gemcitabine reduced the growth rate and final volume of PC cell-derived tumors, the combination of ISG15 knockdown and Gemcitabine treatment (ISG15-sh2+Gemci) showed potent tumoricidal effect compared with the control group (Fig. 4A-C). Also, the significantly reduced tumor weight in the ISG15-sh2+Gemci group when compared with the ISG15-NC+Gemci group indicated that IGS15 confers resistance to Gemcitabine in PC cells (Fig. 4C).

We next conducted immunocytochemistry analysis to detect ISG15, Ki67 and apoptosis in tumor-derived paraffin-embedded sections, leading us to find that the tumor proliferation rate, reflected by Ki67-positive cells, was reduced by ISG15 deprivation (Fig. 4D, E). The rate of apoptosis was the highest in the ISG15-sh2+Gemci group (Fig. 4D, F), again demonstrating that ISG15 promotes PC cell proliferation and confers chemo-resistance to Gemcitabine. Thus, ISG15 plays an important role in PC progression and regulates the response to treatment and may be critical for clinic outcomes.

### Autophagy was impeded after ISG15 knockdown

Autophagy is essential for maintaining cellular homeostasis and survival and does so through the removal and recycling of unwanted cellular materials. ISG15-deficiency has been reported to inhibit autophagy in macrophages 34. Moreover, ISG15 modifies P62 and Beclin 1 in the forms of ISGylation, both of which are important participants of autophagy, and regulates the stability of these proteins 23, 35.

**Figure 5:**
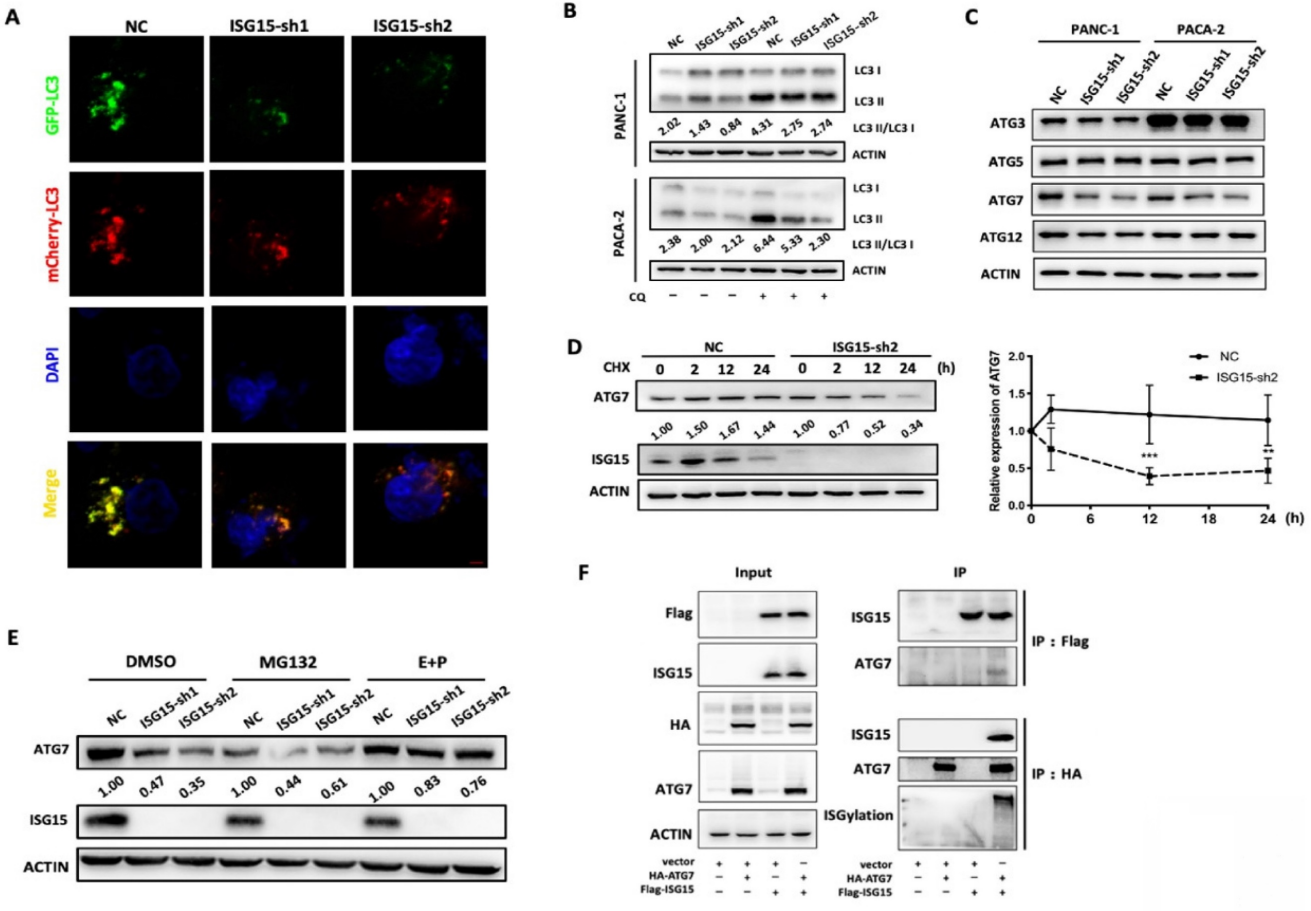
** LC3 conversion was blocked due to destabilization of ATG7 by ISG15 knockdown in PC cells. A,** Confocal microscopic images of GFP-mCherry-LC3-labeled autophagosomes in ISG15-depleted or control PANC-1cells. Images were captured under 1,600× magnification (scale bar: 100μm). **B,** Inhibition of LC3 II degradation in lysosomes by chloroquine (CQ) rescued the decreased LC3 II/I ratio as revealed by Western blotting. **C,** Under the condition of ISG15 knockdown, the expression of LC3-conversion-related molecules (ATG3, ATG5, ATG7 and ATG12) was analyzed with Western blotting. **D,** In Western blotting experiments, the intensity of ATG7 was measured and normalized with ACTIN, and the expression levels relative to that of 0 h are shown. Plots of relative expression level of ATG7 in ISG15-depleted or control cells after Cycloheximide (CHX) treatment are shown. **E,** Control or ISG15-depleted cells were treated with MG132 or E64D/ Pepstatin A (E+P). The grey value of the bands was measured and normalized with the corresponding actin band. Relative expression levels normalized to the intensity of control (NC). **F,** FLAG-tagged ISG15 and HA-tagged ATG7 were overexpressed separately or simultaneously in 293T cells. Co-immunoprecipitation was performed as described in Methods and materials. Representative images are shown. (** *p*<0.01; *** *p*<0.001).

We then investigated whether autophagy was altered in ISG15-depleted PC cells. To visualize autophagosomes, we established a PANC-1 cell line which stably expressed a tandem monomeric mCherry-GFP-tagged LC3B 31. As shown in Fig. 5A and Fig. S3A, the fluorescence dots were reduced when ISG15 was knocked down, indicating a reduction in autophagosomes.

When autophagy is activated, a cytosolic form of LC3 (LC3-I) is conjugated to phosphatidylethanolamine to form LC3-phosphatidylethanolamine conjugate (LC3-II), which is then recruited to autophagosomal membranes and modulates the formation of autophagosomes 36. The level of conversion, shown as LC3-II: LC3-I ratio, can be used to indicate the activation of autophagy 37. The LC3-II: LC3-I ratio was reduced in ISG15-depleted PC cells (Fig. 5B). The reduction of the LC3-II:LC3-I ratio may be caused by either elevated LC3 II degradation or inhibition of the conversion process 37. We found that chloroquine (CQ), an inhibitor of LC3 II degradation in autophagy, failed to rescue the decreased LC3 ratio in ISG15-depleted PC cells. Additionally, the accumulation of LC3 II induced by CQ was lower in ISG15-depleted cells than in control cells, indicating inhibition of autophagy (Fig. 5B). These data suggest that ISG15 depletion blocks the conversion process of LC3 and reduces the formation of autophagosomes, leading to the inhibition of autophagy.

### ISG15 binds to ATG7 and regulates its degradation

The LC3 conversion is induced by several molecules, including ATG3, ATG5, ATG7 and ATG12. We found that the protein level of ATG7, which facilitates LC3-phosphatidylethanolamine, was reduced in ISG15-depleted PC cells (Fig. 5C).

As a type of post-translational protein modifications, ISGylation is reportedly involved in the modification of various proteins and regulates their stability 20. We investigated whether ISG15 can form a complex with ATG7 and regulate its degradation. When protein biosynthesis was blocked by cycloheximide (CHX), the degradation of ATG7 was apparently accelerated in ISG15-depleted PANC-1 cells (Fig. 5D). Moreover, there was no significant change in the mRNA level of *ATG7* after ISG15 depletion (Fig. S3B). These data together suggest that ISG15 regulates the stability of ATG7. We then inhibited two major degradation pathways, the proteasomal degradation and the lysosomal degradation pathways, using their corresponding inhibitors, MG132 and E64D/Pepstatin A (E+P), respectively. Inhibition of the proteasome pathway had no effect on ATG7 stability, while the E+P treatment partially rescued the acceleration of ATG7 degradation mediated by ISG15 knockdown (Fig. 5E). To ask whether ISG15 protein can bind to ATG7, we over-expressed FLAG-tagged ISG15 and HA-tagged ATG7 in 293T cells and found that they co-immunoprecipitated with each other. And the ISGylation of ATG7 increased, when ISG15 and ATG7 were overexpressed simultaneously (Fig. 5F).

Taken together, these data revealed a new function of ISG15 in PC cells. By modulating the expression level of ATG7, ISG15 regulates LC3 I to II conversion, an essential step of autophagy vesicle expansion, and modulates intracellular autophagy. At the mechanistic level, as an ubiquitin-like protein ISG15 forms a complex with ATG7 and stabilizes it. The depletion of ISG15 promotes the degradation of ATG7 through the autophagy-lysosome pathway, leading to the inhibition of LC3 conversion and autophagosome formation, which in turn might avoid excessive reduction of ATG7 protein and maintains intracellular homeostasis.

### ISG15 modulates Gemcitabine sensitivity of PC through autophagy

**Figure 6:**
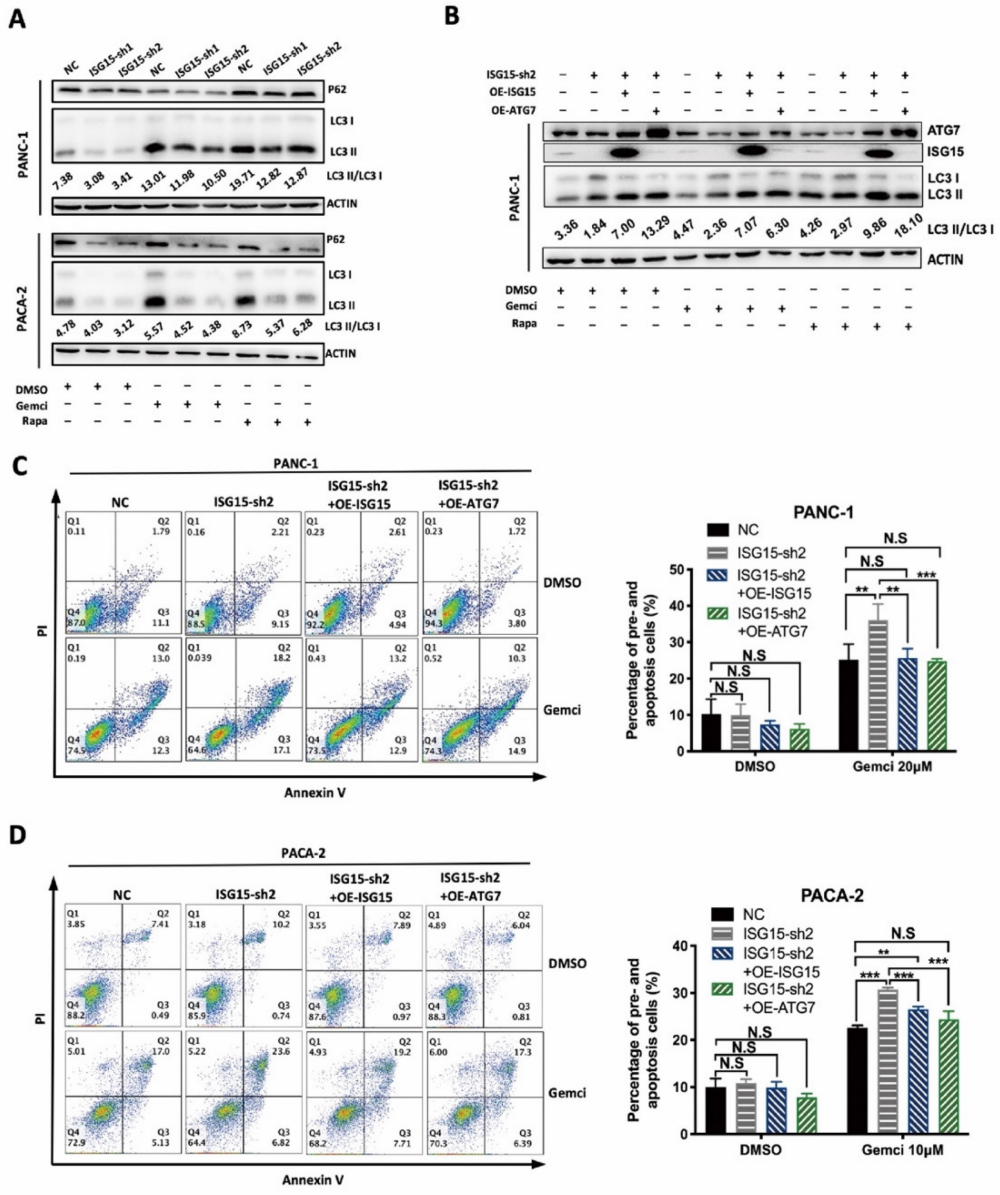
** The autophagy regulated by ISG15 affects the Gemcitabine sensitivity of PC. A,** LC3 II/I ratio was calculated in PC cells treated with DMSO (as a control), Gemcitabine (Gemci) and Rapamycin (Rapa), (as positive inducers of autophagy). LC3 I to II conversion ratio was calculated following the formula: LC3 II/I= (grey value of LC3 II band) / (grey value of LC3 I band), and representative results are shown with the conversion ratio marked.** B-D,** Effects of overexpression of ISG15 or ATG7 in ISG15-silenced PC cells. Effects on LC3 conversion were analyzed with Western blotting. LC3 conversion ratio was calculated as described in the preceding part and noted in the image (B). Apoptosis induced by Gemcitabine was detected with flow cytometry, and the statistical results are shown on the right, with representative pictures shown on the left (C, D). (** *p*<0.01; *** *p*<0.001).

It has been reported that Gemcitabine can induce the increase of autophagy and that the inhibition of autophagy reduces PC cell malignancy and potentiates the tumoricidal effect of Gemcitabine 38, 39. We also found that Gemcitabine treatment increased the LC3-II:LC3-I ratio, similar to Rapamycin (Rapa), an autophagy activator (Fig. 6A).

After ISG15 depletion, the rate of cellular apoptosis was increased after administration of Gemcitabine (Fig. 3D), which was further enhanced by CQ (Fig. S4A and Fig. S4B), suggesting that ISG15 promotes gemcitabine resistance of PC cells through autophagy.

We next used siRNAs to deplete ATG7 and then detected the LC3 I to II ratio in PC cells. Upon ATG7 knockdown, the LC3 I to II conversion was significantly inhibited in PANC-1 cells (Fig. S2F). We also over-expressed ISG15 or ATG7 in ISG15-depleted PC cells and found that ISG15 over-expression rescued the reduced protein level of ATG7 and elevated the LC3-II:LC3-I ratio. While ATG7 over-expression had no impact on the protein level of ISG15, it rescued the reduced LC3-II:LC3-I ratio induced by ISG15 depletion (Fig. 6B).

Finally, we determined whether altered ISG15 and ATG7 levels could change the rate of apoptosis. When ISG15 or ATG7 was over-expressed, cellular apoptosis induced by Gemcitabine was reduced in ISG15-depleted cells (Fig. 6C and Fig. 6D). In addition, the restoration of the levels of ATG7 or ISG15 in ISG15-depleted cells rescued the inhibition of cell proliferation, migration and invasion (Fig. S2G, H), suggesting that ISG15 plays an important role in modulating PC cell malignancy and that ATG7 modulates ISG15.

## Conclusions

In this study, we revealed a new link between ISG15 and autophagy. Through modulation of ATG7 stability, ISG15 maintains proper cellular autophagy both under normal physiological conditions and when Gemcitabine is present, thus promoting the proliferation and survival of PC cells *in vitro* and *in vivo*. These new findings provide new insights into the mechanisms of chemoresistance of PC cells and offer a potential new strategy of suppressing autophagy for sensitizing PC to chemotherapy and ultimately improving the clinical outcomes.

## Discussion

Pancreatic cancer is one of the most lethal cancer types 1. The high mortality rate is mainly due to late diagnosis, early metastasis and limited responses to chemotherapy or radiotherapy. As the standard chemotherapeutics for PC treatment, Gemcitabine presents a moderate effect on ameliorating prognosis. In the past decades, efforts have been focused on the improvement of drug sensitivity through the combined use of potential chemo-sensitizers, such as autophagy inhibitors. Because increasing evidence suggests that autophagy plays an important role in mediating chemoresistance, sensitization of PC to Gemcitabine through autophagy inhibitor has become a promising approach. However, in a phase 2 clinical trial, HCQ, a classic inhibitor of autophagy that functions by intervening the formation of autolysosomes, fails to function as an efficient chemo-sensitizer. As such, new methods of interfering autophagy are needed to sensitize PC to chemotherapy.

In the current study, we found that the expression of ISG15, a ubiquitin-like protein, is elevated in PC tissue sections compared with adjacent tissue. Also, we discovered a link between PC grades and ISG15 expression, which may allow for an earlier diagnosis of PC. However, a larger size of samples, including both tissue biopsies and liquid biopsies, are needed for future analysis to confirm this notion.

ISG15 reportedly serves as a critical microenvironmental factor for PC stem cells 24, and its degradation leads to the suppression of cancer stem cell-like features, including proliferation, migration and tumorigenesis in xenograft tumors induced in nude mice 25, 26. Consistent with these reports, we observed decreased malignant properties of PC cells both *in vitro* and *in vivo* when ISG15 expression was reduced. However, it is not clear how ISG15 promotes tumor-associated phenotypes in our study, nor is it in previous reports. *Yuan et al* showed that ISG15 may play a tumor promoting role via the c-MET/Fyn/ β-catenin pathway with the precise mechanism still undefined 40.

**Figure 7:**
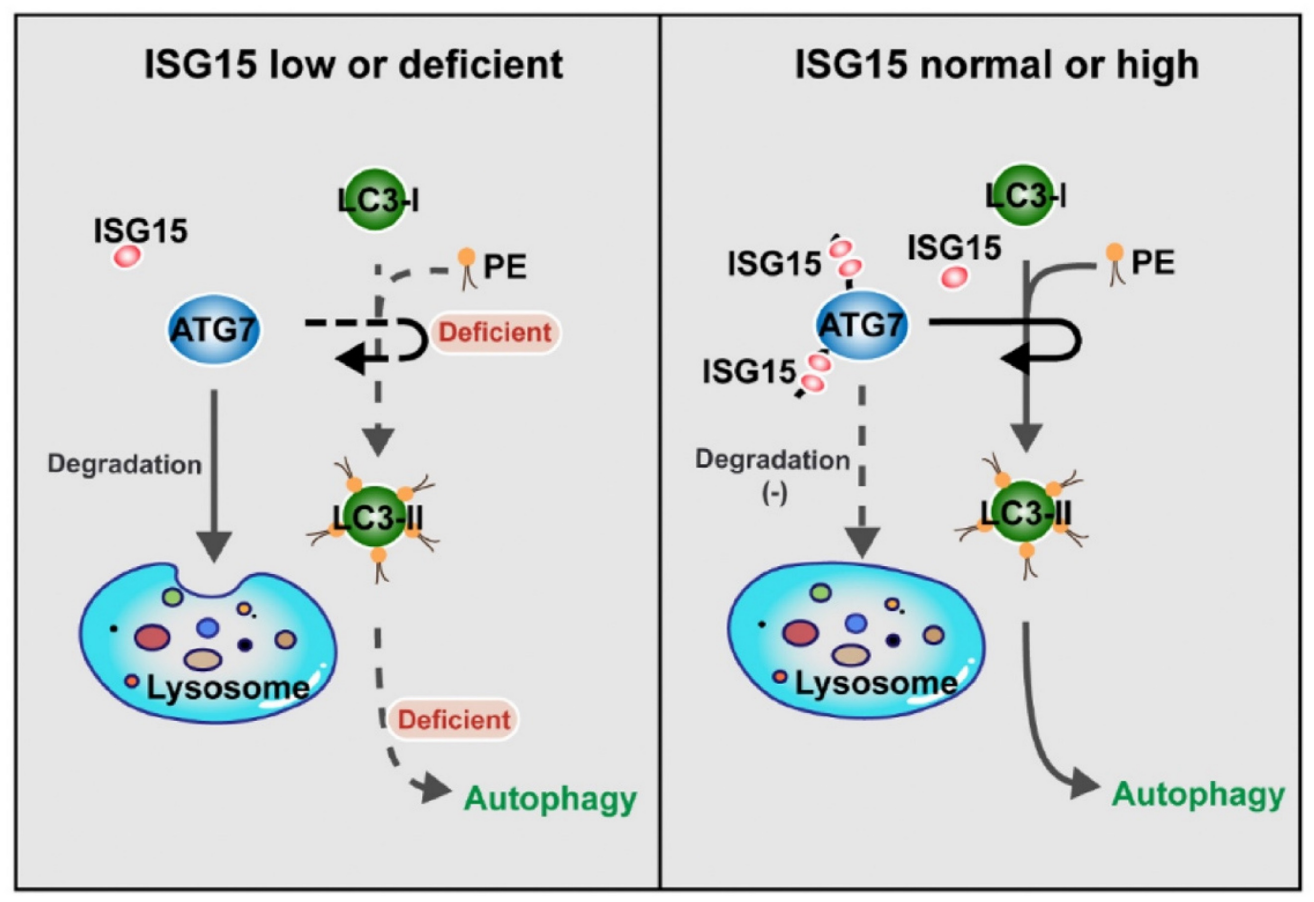
** ISG15 regulates autophagy by modulating ATG7 degradation: the model.** When the expression of ISG15 is low or deficient, ATG7 is degraded by lysosome-mediated pathway, and the transformation from LC3-I to LC3-II is inhibited, with the autophagy level reduced (Left panel). In the cells with normal or high ISG15 expression, ISG15 can inhibit ATG7 degradation and promotes the transformation from LC3-I to LC3-II, resulting in autophagy (Right panel).

ISG15 has been reported as a potential predictor for drug sensitivity 27, 41, and its impact on chemosensitivity has been shown in various cancers with conflicting roles 42-44. In colon cancer, ISG15 reportedly confers chemoresistance to Trametinib 42. However, according to the studies by *Jeon et al* and *Wang et al*, ISG15 enhances the tumoricidal effect of chemotherapeutics, which is mediated by ISGylation of essential molecules and inhibition of their translocation or recruitment to their respective normal functional locations 43, 44. In our study, we found that ISG15 modulates the stability of ATG7 and confers resistance to Gemcitabine in PC cells. ISGylation of ATG7 reduces its degradation in lysosomes, thus stabilizing autophagosome, because ATG7 is a key protein of autophagosomal elongation. The lack of ISG15 destabilizes ATG7, impedes LC3 conversion, and increases the sensitivity to Gemcitabine while the restored expression of ATG7 reverses the increased sensitivity by reestablishing autophagosome formation (Fig. 7). These results also provide mechanistic explanations for the phenotypes caused by ISG15 knockdown, because autophagy plays important roles in maintaining malignant properties of cancer cells.

Although the interplay between ISG15 and the autophagy machinery has been reported in several studies, the conclusions are conflictive. During cellular defense responses, ISG15 is colocalized with P62 and promotes autophagosome and lysosome fusion 35. However, during long-term treatment of interferons, ISGylation of Beclin 1, an inducer of ISG15 expression, inhibits autophagy by attenuating Beclin 1 ubiquitination 23. More recently, *Kong et al* reported that the deletion of ATG7 or ATG5 induces ISG15 expression through the STING pathway, which is activated by accumulated cytosolic dsDNA caused by ATG5/7 depletion. Also, regulation of autophagy by rapamycin treatment or serum deprivation can also upregulate ISG15 45. Combining these earlier studies with our current study, we postulate that ISG15 may serve as a positive regulator of the autophagy process. In this scenario, ISG15 stabilizes ATG7 and safeguards autophagy, while autophagy is elevated in response to Gemcitabine to maintain intracellular homeostasis. If ISG15 expression is reduced, the remaining function of autophagy cannot meet the challenge of Gemcitabine, leading to the reduction of cellular apoptosis. Reduced ISG15 level leads to impeded function of autophagy, which may in return assist in attenuating the degradation of ATG7, which occurs in lysosomes and may be mediated by the formation of autolysosomes in late stage of autophagy. This feedback mechanism maintains the residual function of autophagy. Additional experimentation is clearly needed to further unveil the relationship between ISG15, autophagy and the treatments of PC.

Our current study is mainly focused on the role of ISG15 as a conjugated protein with its targets, and the function of ISGylation as a free unconjugated protein is poorly depicted. As an extracellular molecule, ISG15 is reportedly secreted by tumor-associated macrophages (TAM) and contributes to CSC phenotypes 24. In melanoma cells, ISG15 secreted by melanoma cells modulates the phenotype of tumor-infiltrating dendritic cells (DCs) by inducing the expression of E-cadherin, resulting in the impairment of DCs and cancer cell escape 46. *Burks et al* found that the depletion of ISG15 downregulates PDL-1 expression, increases tumor-infiltrating CD8^+^ lymphocytes, and promotes tumor cell apoptosis 25. These findings point to the immunomodulatory properties of ISG15 and its potential as an immune adjuvant in cancer therapies. Whether the immunomodulatory properties of ISG15 can prove to be beneficial for PC therapy merits future investigation.

## Supplementary Material

Supplementary figures.

## Figures and Tables

**Table 1:** Primers

Name	Sequence (5'-3')
*ATG7-*forward	ATGATCCCTGTAACTTAGCCCA
*ATG7-*reverse	CACGGAAGCAAACAACTTCAAC
*β-ACTIN*-forward	TCCTGTGGCATCCACGAAACT
*β-ACTIN*-reverse	GAAGCATTTGCGGTGGACGAT

## Abbreviations

PC: Pancreatic cancer
ISG15: Interferon-induced gene 15
ATG7: Autophagy-related gene 7
GEO: Gene Expression Omnibus
CQ: Chloroquine
LC3-I: cytosolic form of LC3
LC3-II: LC3-phosphatidylethanolamine conjugate
CHX: Cycloheximide
E+P: E64D/Pepstatin A
Gemci: Gemcitabine
