# Supplementary material for: ISG15 Promotes Progression and Gemcitabine Resistance of Pancreatic Cancer Cells Through ATG7
Source: Int J Biol Sci. 2024 Jan 21;20(4):1180–93. doi: 10.7150/ijbs.85424 (PMC10878160; doi:10.7150/ijbs.85424)
Supplement: Supplementary file 1 — Supplementary figures. [file ijbsv20p1180s1.pdf]

Meng YL` Revised Supplementary Figures and Legends

Fig. S1

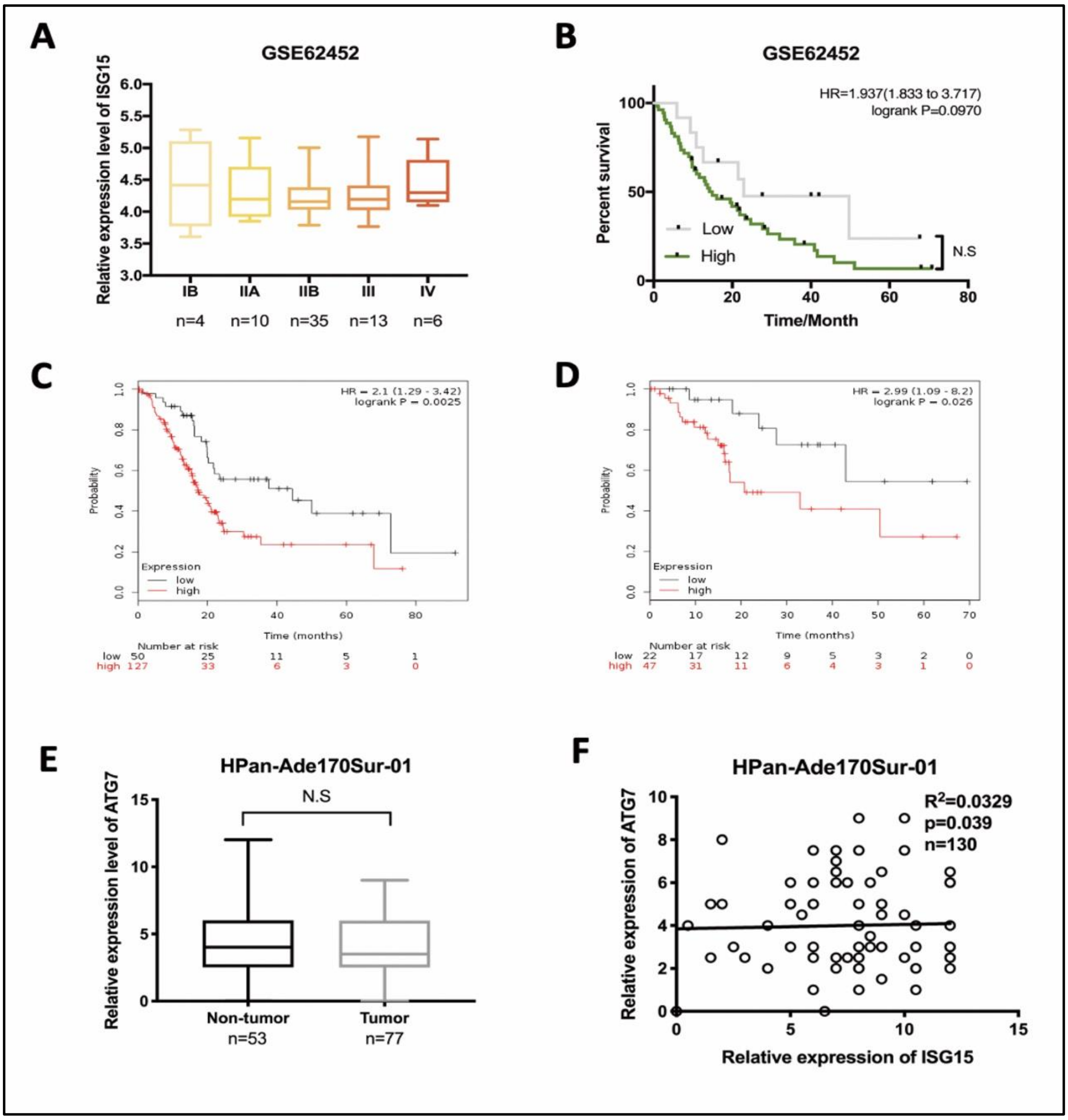

Fig. S1. A, ISG15 expression exhibited no significant correlation with the PC stage. Data from dataset GSE62452 were analyzed. **B,** No significant correlation between ISG15 and PC patient survival was shown based on the analysis of GSE62452 dataset. **C-D,** K-M survival analysis was used to analyze the correlation between overall survival (C) and recurrence-free survival (D). **E,** Box-plot of the expression of ATG7 in tumor and adjacent non-tumor tissue sections from PC microarray. Method of H-score evaluation was described in Methods and Materials. **F,** Analysis of the correlation between the expression levels of ISG15 and ATG7 of PC microarray.

Fig. S2

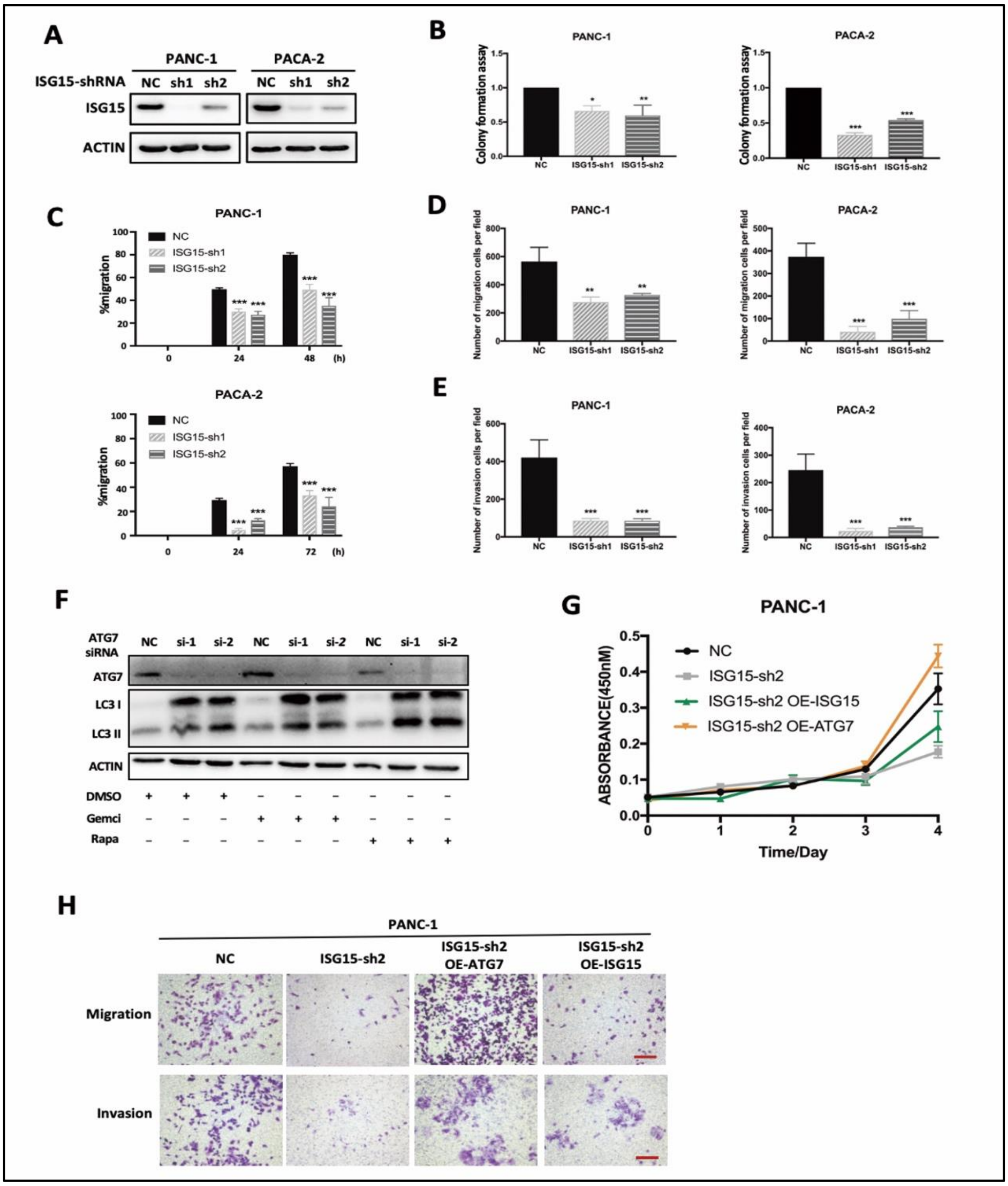

Fig. S2. A, ISG15-targeted shRNA significantly reduced ISG15 in both PANC-1 and PACA-2 cells. Representative results of Western blotting are shown. **B-E,** Statistical results of colony formation assays (B), wound healing assays (C), chamber migration (D) and chamber invasion (E) are shown. **F,** Effects of ATG7 knockdown on LC3 II/I ratio. 48h after transfection of ATG7-targeted siRNA, DMSO (as the control), Gemcitabine (Gemci) or Rapamycin (Rapa) was added. **G-H,** Restored expression of ISG15 or ATG7 rescued the effects on cell proliferation (G), migration and invasion (H) in ISG15-depleted cells (scale bar; 50 μm). (* $p<0.05$; ** $p<0.01$; *** $p<0.001$).

Fig. S3

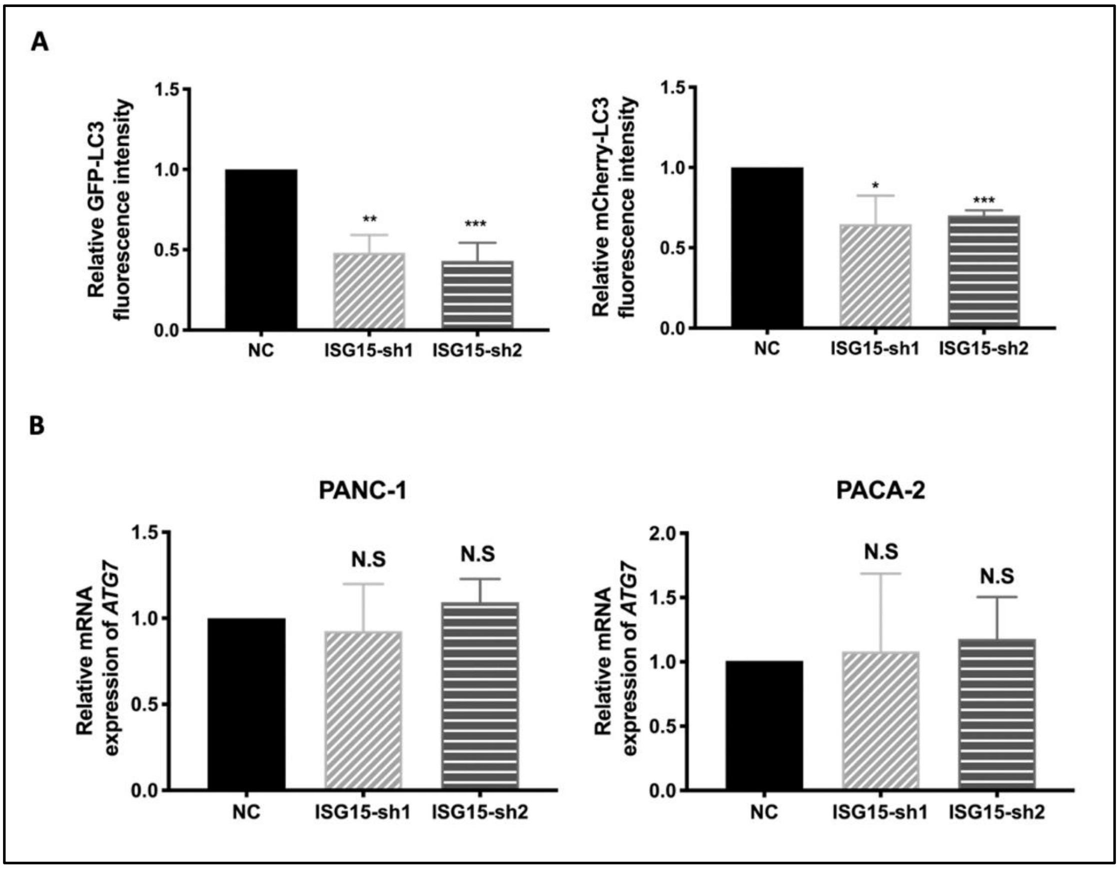

Fig. S3. A, Relative fluorescence intensity for the confocal microscopic images of GFP-mCherry-LC3-labeled autophagosomes in ISG15-depleted or control PANC-1cells. **B,** Relative mRNA levels of *ATG7* were assayed by RT-PCR in PC cells with or without ISG15 depletion. *ACTIN* was used as an internal loading control. (* $p<0.05$; ** $p<0.01$; *** $p<0.001$).

Fig. S4

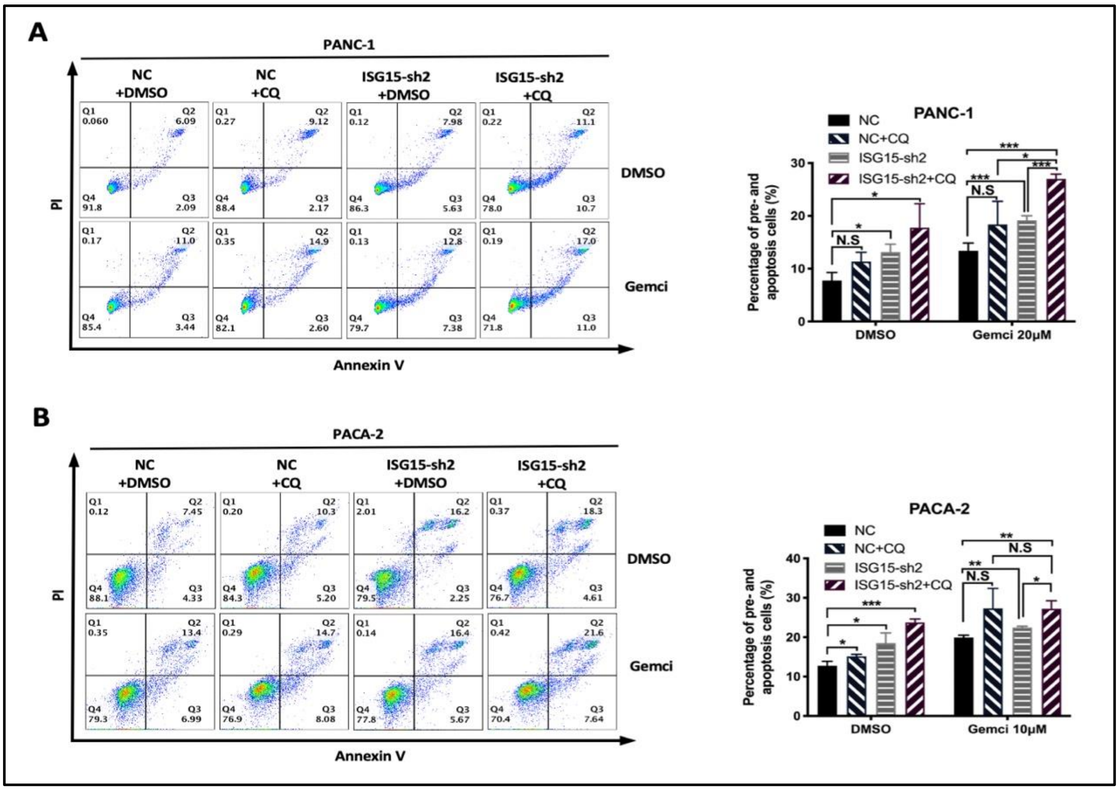

Fig. S4. A-B, Apoptosis induced by Gemcitabine (Gemci) or/and chloroquine (CQ) in PANC-1(A) and PACA-2 (B) cells with or without ISG15 depletion was detected with flow cytometry, and the statistical results are shown on the right, with representative pictures shown on the left. (* p<0.05; ** p<0.01; *** p<0.001).
